# Does Developmental Timing Matter? Comparative Analysis of Day 5 and Day 6 Euploid Blastocyst Transfers in Recurrent Implantation Failure Patients

**DOI:** 10.3390/biomedicines13112741

**Published:** 2025-11-10

**Authors:** Alper Şişmanoğlu, Süleyman Cemil Oğlak, Zafer Atayurt, Fulya Gökdağlı Sağır, Ulun Uluğ

**Affiliations:** 1 Department of Obstetrics and Gynecology, Altınbaş University Faculty of Medicine, 34147 Istanbul, Türkiye; alper.sismanoglu@altinbas.edu.tr; 2 Department of Obstetrics and Gynecology, Health Sciences University, Gazi Yaşargil Training and Research Hospital, 21500 Diyarbakır, Türkiye; 3 Department of Embryology, Kolan International Hospital, 34384 Istanbul, Türkiye; zaferatayurt@gmail.com; 4 Department of Obstetrics and Gynecology, Kolan International Hospital, 34384 Istanbul, Türkiye; docflyster@gmail.com; 5 Department of Obstetrics and Gynecology, Haliç University Faculty of Medicine, 34060 Istanbul, Türkiye; uulug@hotmail.com

**Keywords:** delayed blastocyst development, frozen–thawed euploid blastocyst transfer, in vitro fertilization outcomes, recurrent implantation failure, maternal age, PGT-A

## Abstract

**Background/Objectives**: The timing of blastocyst formation is an important factor in in vitro fertilization (IVF) outcomes. While many studies have shown similar pregnancy rates for euploid blastocyst transfers occurring on day 5 or 6, controversy remains, especially regarding patients with recurrent implantation failure (RIF). This study aimed to evaluate whether day 5 (D5) and day 6 (D6) euploid blastocysts differ in terms of the clinical outcomes achieved after frozen–thawed euploid embryo transfer, with subgroup analysis by maternal age in RIF patients. **Methods**: This retrospective analysis included a cohort of 514 patients who experienced RIF, categorized into two distinct groups: the initial group consisting of individuals who underwent embryo biopsy on day 5 (*n* = 456 euploid transfers) and the subsequent group comprising patients who underwent biopsy on day 6 (*n* = 58 euploid transfers) following ovum retrieval. These groups were utilized based on the completion of blastocyst development and the eventual pregnancy outcomes after the first post-frozen–thawed euploid embryo transfer confirmed by PGT-A. **Results**: Out of the total cohort of 514 RIF patients, 472 individuals were younger than 40 years of age, representing 91.8% of the sample population. The IVF process yielded successful positive pregnancy outcomes in 85.4% of the cases (439 out of 514)., and there was no statistically significant difference in maternal ages between the day 5 and day 6 biopsy cohorts (*p* = 0.286). The proportion of successful clinical pregnancies per transfer was markedly higher among those who underwent biopsy on day 5 in comparison to those subjected to biopsy on day 6 (79.2% vs. 58.6%, *p* = 0.002). In patients aged 35 years and younger who underwent biopsy on day 5, the rates of successful clinical pregnancy outcomes were superior to those who underwent biopsy on day 6 (79.8% vs. 56.5% *p* = 0.016). In individuals older than 35 years of age, no significant discrepancies in clinical pregnancy rates were observed between the two groups. **Conclusions**: Day 6 euploid embryo transfer was associated with lower clinical pregnancy rates per transfer compared with those on day 5, particularly in patients younger than 35 years of age. In women ≥ 35 years of age, outcomes did not differ significantly. These outcomes suggest that developmental timing interacts with maternal age in determining clinical outcomes. Further prospective studies with larger and more balanced cohorts are needed to confirm these findings.

## 1. Introduction

In vitro fertilization (IVF) represents a multifaceted procedure predominantly utilized to aid couples struggling to achieve natural conception, with the supplementary objective of mitigating the occurrence of genetic disorders [1]. Generally, the entire IVF regimen spans approximately three weeks; however, this timeframe might fluctuate and, at times, be segmented into smaller phases owing to varying stimulation protocols [2]. As advancements in IVF technology persist, several determinants are recognized as pivotal contributors to the effectiveness of the treatment, such as the duration of infertility experienced by the individual, lifestyle choices, the methodology employed for oocyte retrieval, the endometrial thickness, the quantity of embryos transferred, and the quality of the resultant blastocysts [3]. Numerous elements impact the process of embryogenesis, which can originate from either male or female factors and can significantly influence the developmental trajectory of the embryo [4,5]. The literature has documented certain distinctions between intracytoplasmic sperm injection (ICSI) and traditional in vitro fertilization (IVF) methodologies in the initial stages of embryo development [6]. The blastocyst stage of embryo development is distinguished by active cellular division and the process of cellular differentiation [7]. A variety of staging systems exist that can be utilized to identify mature embryos [8]. The blastocyst stage is characterized by the presence of a fluid-filled cavity termed a blastocele, in addition to the differentiation of the outer trophoblast layer and the inner cell mass; these defining characteristics can typically be observed on the fifth day of embryonic development [9]. A delayed advancement to the blastocyst stage in embryos might serve as an indicator of their developmental competency. In most instances, the majority of embryos attain the blastocyst stage within five days; however, certain embryos might necessitate an extended duration of six or seven days to reach this developmental milestone. Such delays in embryonic development might be attributed to various factors that influence the embryo’s capacity for development, including maternal age and oocyte quality. It is well-established that fresh day 5 transfers yield more favorable outcomes compared to those conducted on day 6 [10]. This disparity in pregnancy outcomes has been primarily ascribed to the asynchrony observed between the endometrium and day 6 embryos. Efforts to rectify this asynchrony by freezing all embryos and subsequently transferring them alongside synchronized endometrial preparation have yielded inconsistent outcomes, as reported in the existing literature. A meta-analysis conducted by Sunkara et al. indicated that if a day 6 embryo achieves a developmental level comparable to that of a day 5 embryo, analogous pregnancy outcomes might be attained in cycles involving frozen–thawed embryo transfers [11]. In contrast to this, Ferreux et al. published outcomes indicating that pregnancy outcomes are superior when transferring embryos frozen on day 5 in comparison to those frozen on day 6 [12]. There exist a paucity of data regarding the implications surrounding euploid embryos on days 5 or 6 when conducting frozen–thawed embryo transfer. The cryopreservation of blastocysts followed by their subsequent transfer post-thawing affords the opportunity to perform a trophectoderm biopsy, thereby enabling genetic assessment of the cryopreserved embryos and the transfer of euploid embryos. The extant literature fails to yield conclusive evidence regarding the influence of late embryonic development on euploid embryo pregnancy success rates. The aim of our investigation is to evaluate the influence of the duration of embryonic development on the probability of successful clinical pregnancy establishment in patients with RIF via in vitro fertilization and frozen–thaw embryo transfer subsequent to preimplantation genetic testing for aneuploidy (PGT-A).

## 2. Materials and Methods

This retrospective analysis was conducted using the data of patients experiencing recurrent implantation failure (RIF) at a private hospital IVF center from the years 2019 to 2023. A total of 514 patients’ first embryo transfer cycles were included in this study; among them, 456 had embryos biopsied on day 5, and 58 on day 6. No a priori power calculation was performed, as this was a retrospective analysis of all eligible cases during the study period. Recurrent implantation failure was operationally defined as the occurrence of at least two unsuccessful IVF cycles involving high-quality embryos, taking into account a cumulative implantation chance of 60% as the threshold for initiating further investigations [13]. All subjects presented with a normal karyotype devoid of both single-gene and numerical chromosomal anomalies. Patients exhibiting severe male-factor infertility, endometrial factors, or uterine malformations were excluded from the study sample, and only those who possessed at least one euploid embryo following embryonic biopsy for aneuploidy screening were included in the analysis. The cohorts were stratified based on the completion of blastocyst development on either day 5 or day 6. Patients who had good-quality embryos for biopsy on both the 5th and 6th days were excluded to avoid intra-patient bias. A comparative analysis was conducted between these two groups, focusing on variables such as age, oocyte count, fertilization status, the number of embryos achieving blastocyst stage, and the quantity of cryopreserved embryos that underwent genetic analysis via trophectoderm (TE) biopsy. On the 2nd or 3rd day of the menstrual cycle, ovarian stimulation was commenced utilizing recombinant follicle-stimulating hormone (rFSH) either in isolation or in combination with human menopausal gonadotropin (hMG). The daily dosage was modulated between 225 IU and 450 IU in accordance with the ovarian reserves of the patient. To suppress luteinizing hormone, 10 mg of Medroxyprogesterone acetate (Tarlusal 5 mg tablet, Deva Holding A.Ş, Istanbul, Turkey) was administered daily from the onset of ovarian stimulation across all cycles within this study. Recombinant human chorionic gonadotropin (Ovitrelle 250 micrograms, Merk Serono, Modugno (BA)/Italy) was administered 35 to 36 h prior to the ovum retrieval procedure to facilitate the final maturation of the follicles.

### 2.1. Embryo Culture, TE Biopsy and Next-Generation Sequencing

The retrieved oocytes were cultured before fertilization in Quinn’s Advantage Fertilization Medium (Sage Bio-Pharma, Inc., Trumbull, CT, USA) with 15% serum protein substitute (SPS) (Sage BioPharma, Inc., Pasadena, CA, USA) in a triple gas phase of 5% CO_2_, 5% O_2_, and 90% N_2_. The insemination, or intracytoplasmic sperm injection (ICSI), was performed 38–41 h after antagonist protocol administration. The intracytoplasmic sperm injection (ICSI) technique is routinely employed as the standard fertilization method within our laboratory, regardless of the underlying infertility etiology, and was implemented for all patients in this study to minimize fertilization failure. Following ICSI, all embryos were further cultured in microdrops (Quinn Advantage Cleavage Medium (Sage BioPharma, Inc.)) with 15% SPS under low oxygen (5% O_2_) until day 3. At 70–72 h after insemination/ICSI, all cleaved embryos were further cultured in microdrops of (Quinn Advantage Blastocyst Medium (Sage BioPharma, Inc.)) with 15% SPS to reach the blastocyst (day 5/6) stage for trophectoderm (TE) biopsy. Laser-assisted hatching was performed on day 4. To evaluate the embryonic quality and identify the optimal embryo(s), we adhered to the guidelines established during the 2011 ESHRE Istanbul Consensus workshop on embryo assessment [14]: only the expanded blastocysts considered to be of a desirable quality (4, 5, 6, AB, BA, and BB) with diameters of more than 150 μm were defined as qualified blastocysts and were suitable for TE biopsy. The day 5 blastocyst stage without expansion (<150 μm) was further cultured until day 6, and TE type A or B biopsy was performed on day 6 expanded blastocysts with an ICM. The biopsied TE cells were immediately placed in an RNAse–DNAse-free polymerase chain reaction tube and amplified using the SurePlex DNA Amplification System (Illumina, Inc., San Diego, CA, USA). Extracted cells were placed in 2 μL of buffer and sent frozen to Geneticiks Genetic laboratory for PGT-A using a high-resolution, next-generation sequencing (hr-NGS) platform. The Thermo Fisher Scientific “ReproSeq™ PGS Kit” was in the genetic laboratory to detect genetic abnormalities in embryos. With the help of PGT-A, we detected euploid and ruled out aneuploid and mosaic embryos.

### 2.2. Vitrification–Warming Protocol

The biopsied blastocysts were vitrified 2–4 h after TE biopsy, and the vitrification and warming protocols were conducted with Cryotech (Repro-Support Medical Research Centre, Co. Ltd., Tokyo, Japan). Euploid embryo/s was/were selected for transfer and warmed. Embryos were cultured in a blastocyst medium at 37 °C (5% CO_2_ and 5% O_2_) for 1–2 h before transfer. At the time of embryo transfer, we checked the survival of warmed blastocysts, which was defined as >80% of cells being intact and full re-expansion. In this study, all of the warmed blastocysts survived with full re-expansion.

Upon receipt of the genetic analysis results, only chromosomally normal (euploid) embryos were selected for transfer following endometrial preparation utilizing 6–8 mg of oral Estradiol tablets, and patients were administered either 100 mg of intramuscular progesterone in oil or 600–800 mg of micronized progesterone suppositories five days prior to the transfer of euploid embryos. For all patients with euploid embryos, transfer occurred five days after initiating progesterone supplementation, irrespective of the biopsy day. A transabdominal ultrasound was employed to accurately guide all transfer procedures. A blood hCG level exceeding 5 mIU/mL, measured 11 to 12 days post-embryo transfer, was deemed indicative of a positive pregnancy outcome. The clinical pregnancy rate was defined as the presence of a viable intrauterine gestational sac with fetal heartbeat at the 8th week of gestation. The pregnancies that potentially would not reach the 8th week of gestation were categorized as miscarriages. In this study, the pregnancy outcomes were reported for each transfer regardless of the number of embryos transferred, and for each patient, only one embryo transfer procedure was included in this study. The characteristics of the IVF outcomes for the two groups are succinctly summarized in Table 1. All participants provided their informed consent and ethical approval was obtained from the Altinbas University Clinical Studies Ethics Committee (File number: 2025-005). This approval was retrospective and in line with national regulations for retrospective studies.

## 3. Statistical Analysis

The statistical software SPSS version 25.0 (IBM Corporation, Armonk, New York, NY, USA) was employed to examine the variables under consideration. We used the Shapiro–Wilk test to assess the data’s adherence to a normal distribution. The Mann–Whitney U test, enhanced with Monte Carlo simulations, was employed to compare variables such as age, the duration from oocyte collection to transfer, the number of oocytes, metaphase status, fertilization rates, and the quantity of embryos transferred on the fifth and sixth days in relation to the pregnancy outcome. Data were expressed as median values and are presented in Table 1 and Table 2 to facilitate comparison between the two groups. A comparative analysis of embryo transfers on days 5 and 6 was executed through the Mann–Whitney U test coupled with Monte Carlo outcomes. Pearson’s Chi-Square Monte Carlo Simulation methodology was utilized to evaluate the comparative analysis of overall and stratified age, ongoing pregnancy status, and the day of embryo transfer in conjunction with the pregnancy outcome; comparisons of column ratios were articulated as Benjamini–Hochberg corrected *p*-values. The sensitivity and specificity ratios pertaining to the correlation between actual classifications and classifications derived from the cut-off value, as determined by age and the number of embryos transferred on the biopsy day, were scrutinized and depicted through ROC (Receiver Operating Characteristic) curve analysis. Comparisons between groups were performed using Mann–Whitney U and Chi-Square tests with Monte Carlo simulation. ROC curve analysis was used to assess the discrimination capacity by age threshold (35 years). Drawing on the findings of this analysis, we employed odds ratios accompanied by 95% confidence intervals to elucidate the likelihood of pregnancy based on biopsy day using the positive pregnancy indicators of categorized numbers of embryo transfers and age. Quantitative variables were delineated as mean (standard deviation) and median (1st Quartile/3rd Quartile) in tabular format, while categorical variables were represented as *n* (%). The variables were subjected to analysis at a 95% confidence level, with *p* < 0.05 deemed statistically significant.

## 4. Results

**Figure 1:**
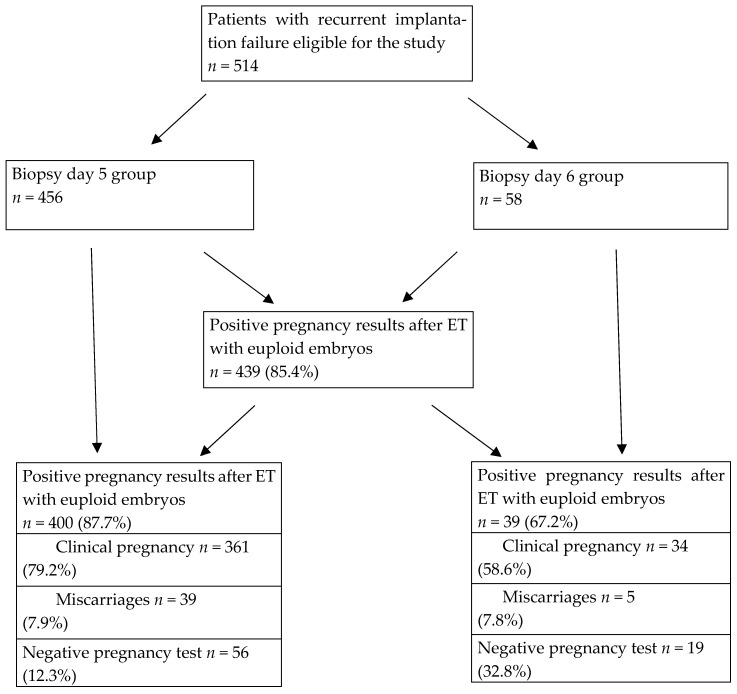
Flow chart of the patients according to pregnancy outcomes.

**Figure 2:**
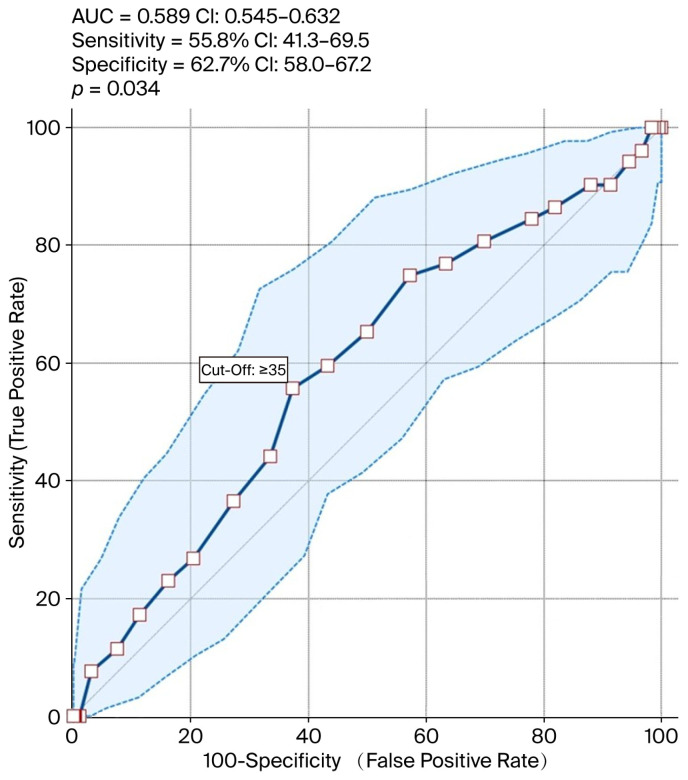
Sensitivity and specificity of ROC prediction model according to age.

Among the total cohort of 514 patients, 472 individuals (91.8%) were aged 40 years or younger. The application of in vitro fertilization (IVF) resulted in positive pregnancy outcomes in 85.4% (439/514) of cases. Statistical analysis indicated no significant difference between the day 5 and 6 biopsy cohorts with respect to maternal age (*p* = 0.286). Furthermore, the incidence of clinical pregnancies was markedly elevated in patients who underwent biopsy on day 5 in comparison to those on day 6 (79.2% vs. 58.6%, *p*= 0.002) (OR %95 CI 2.6 (1.4–4.7)). Figure 1 shows the patient flow diagram of the two groups (day 5 and 6 biopsy) according to the pregnancy outcomes after euploid embryo transfer. All of the outcomes were per embryo transfer and only the first transfers were included. Additionally, the likelihood of achieving a successful clinical pregnancy was found to be 2.6 (OR:1.4–4.7) times greater for individuals who underwent a biopsy on day 5 compared to day 6. No significant discrepancies were observed between the groups regarding oocyte count, number of mature oocytes, fertilized oocyte count, and the number of cryopreserved embryos post-trophectoderm biopsy. The cohort undergoing day 5 biopsy exhibited a greater frequency of single-embryo transfers compared to the day 6 biopsy cohort (74.5% vs. 46.5% *p* = 0.002) (OR %95 CI 2.5 (1.4–4.5)) (Table 2). This variation could be attributed to either patient preference or the quantity of chromosomally normal embryos available. According to the IVF regulations in our country, patients with more than two unsuccessful embryo transfer attempts are allowed to have two embryos transferred in a subsequent cycle if feasible. We observed that transferring more than one embryo did not improve pregnancy outcomes in patients with slow-developing embryos (day 6 biopsy group), as this group did not reach the pregnancy rates achieved in those with embryos that developed on time (day 5 biopsy group). For this reason, we did not exclude these patients from the study, since this subgroup allowed us to emphasize the importance of timely day 5 blastulation. The rates of successful clinical pregnancies were significantly higher among patients aged 35 years and younger who underwent biopsy on day 5 than in those on day 6 (79.2% vs. 58.6%, *p* = 0.016) (OR %95 CI 3.0 (1.3–7.3)). Additionally, the likelihood of achieving pregnancy success in this subgroup was threefold greater. In individuals over the age of 35, no significant differences in clinical pregnancy rates were observed; moreover, the rates of successful pregnancies were considerably higher in the cohort undergoing biopsy with single-embryo transfer on day 5 compared to the group on day 6 (76.1% vs. 48.1%, *p* = 0.04) (OR %95 CI 3.2 (1.5–7.1)). Furthermore, the probability of pregnancy success in this context was 3.2 (odds ratio:1.5–7.1) times higher. Table 3 provides a summary of the analysis of all variables in accordance with the 35-year age threshold. In Figure 2, ROC (Receiver Operating Curve) analysis demonstrates the sensitivity and specificity ratios, with the cut-off values calculated according to age >35 years and biopsy day. The discriminative ability was modest, with an AUC (95% CI) of 0.589 (0.545–0.632); the sensitivity was 55.8% (95% CI: 41.3–69.5); and the specificity was 62.7% (95% CI: 58.0–67.2). These values are presented descriptively and are not utilized for inferential testing.

## 5. Discussion

Factors influencing the success of in vitro fertilization (IVF) encompass endometrial thickness, body mass index, advanced maternal age, recurrent implantation failure, significant male-factor infertility, history of recurrent miscarriage, embryo quality, embryo age, embryo morphology, and genetic characteristics. The critical role of embryo quality in relation to the determinants affecting the success of in vitro fertilization is unequivocal. Embryologists and clinicians continue to encounter challenges in selecting the embryo that possesses the highest probability of successful implantation. Optimizing embryo selection in patients with recurrent implantation failure (RIF) holds substantial clinical significance. Identifying the most developmentally competent embryo—preferably one that achieves blastulation on day 5—can enhance implantation potential and improve overall treatment outcomes. The timing of blastocyst formation serves as an indirect indicator of embryonic viability and chromosomal competence; thus, prioritizing day 5 euploid embryos when they are available may reduce the likelihood of repeated implantation failure. Moreover, integrating morphokinetic parameters and preimplantation genetic testing (PGT-A) into the selection process allows for a more individualized and evidence-based approach. Such strategies not only improve success rates but also minimize emotional and financial burdens for RIF patients by reducing the number of unsuccessful transfers and unnecessary treatment cycles. In our investigation, the pregnancy success rate was markedly diminished when day 6 in comparison to day 5 euploid embryos were transferred. There exists a lack of consensus within the scientific literature regarding the clinical efficacy of biopsy procedures performed on day 5 vs. day 6 embryos. It is already established that euploidy rates decline when embryos are biopsied on day 5, day 6, or day 7 (55.6%, 39.7%, and 27.1%, *p* < 0.001, respectively) [15]. A diminished capacity for blastocyst development and a delay in the onset of the initial cleavage event might be potential explanations for such an outcome.

Investigations concerning the discrepancies between the transfer of embryos at the blastocyst stage on day 5 vs. 6 reveal significant variations in gestational outcomes [16]. Generally, embryos transferred on day 5 have been demonstrated to exhibit superior implantation, clinical pregnancy, and live birth rates. Conversely, embryos transferred on day 6 display reduced rates of implantation and clinical pregnancy, and are also noted to have elevated rates of miscarriage and early pregnancy loss [17,18]. Nevertheless, other studies have reported no significant differences [19,20]. Research conducted by Le Duc Thang et al. illustrates that blastulation timing and embryo quality are pivotal factors in ascertaining the success of single-euploid blastocyst transfers. Specifically, the study indicates that the transfer of day 5 in comparison to day 6 euploid embryos is correlated with higher live birth and ongoing pregnancy rates (38.71% vs. 55.04%, *p* = 0.001) [21]. Na Li et al. noted that day 5 embryos exhibited elevated rates of euploidy; however, this was exclusively applicable to women under the age of 35 [22]. Correspondingly, our study demonstrated that those over the age of 35 had a significantly reduced probability of achieving a successful pregnancy. The literature suggests that older women face an augmented risk of encountering pregnancy complications and adverse birth outcomes during in vitro fertilization procedures. It has been evidenced in academic sources that this phenomenon might be attributed to the increasing percentage of aneuploidy as the duration of blastulation extends [23]. Tiegs et al. established that day 7 euploid embryos can attain comparable and consistent implantation rates relative to day 5 and day 6 euploid blastocysts, provided that blastocyst morphological quality and endometrial–embryo synchrony are appropriately managed [24].

Nevertheless, this study faced certain potential limitations, including the retrospective nature of the data analysis and the relatively constrained sample size of day 6 embryos, which might compromise the statistical validity of the findings. Logistic regression or propensity score analysis potentially cannot be performed due to dataset limitations, nor can we calculate the implantation rates, as data were collected per transfer rather than per embryo and the differences in single- vs. double-embryo transfers between groups might have influenced the outcomes. A further limitation is that we lack the data for live birth rates. Multiple pregnancy and implantation rates were not recorded, and our primary goal was to assess the clinical pregnancy rates, defined by positive heartbeat at the 8th week of pregnancy, after the euploid transfer of embryos biopsied on the 5th or 6th day, thus defining the pregnancy potential of the slow-growing euploid embryos. Consequently, embryos subjected to biopsy on the sixth day exhibited a comparatively less favorable prognosis regarding pregnancy success in contrast to those biopsied on the fifth day [25]. Moreover, it is imperative to acknowledge the potential inconsistencies that might exist in the classification of embryo maturation as well as the laboratory protocols across different clinical environments and research studies. Additional research employing larger sample sizes and more homogeneous cohorts might facilitate a more conclusive understanding.

## Figures and Tables

**Table 1:** Characteristics of IVF outcomes between day 5 and 6 groups according to the oocyte yields and embryos sent for PGT-A.

Parameter	Biopsy Day 5 (*n* = 456)	Biopsy Day 6 (*n* = 58)	*p*-Value
Age ≤ 40 (%) (*n* = 472)	421 (92.4)	51 (88.5)	0.286 ^f^
Age > 40 (%) (*n* = 42)	35 (7.6)	7 (11.5)	
Oocyte number (median Q1–Q3)	11 (7–19)	14.5 (6.5–25)	0.398 ^U^
MII oocytes (median Q1–Q3)	9 (6–18)	13 (5–19)	0.369 ^U^
Fertilized oocytes (median Q1–Q3)	8 (5–13)	10.5 (5–15.5)	0.446 ^U^
Embryos sent for genetic analysis (median Q1–Q3)	4 (3–8)	4 (3–8)	0.514 ^U^

^f^ Fisher Freeman Halton Test (Monte Carlo), ^U^ Mann—Whitney U test (Monte Carlo).

**Table 2:** Pregnancy outcomes after euploid embryo transfers between day 5 and 6 embryo biopsy groups.

Outcome	Biopsy Day 5 (*n* = 456)	Biopsy Day 6 (*n* = 58)	*p*-Value	Odds Ratio (95% CI)
Clinical pregnancy rate *	361 (79.2%)	34 (58.6%)	**0.002** ^c^	2.6 (1.4–4.7)
Miscarriage rate	39 (7.9%)	5 (7.8%)	0.638 ^c^	NS
Single-embryo transfer	340 (74.5%)	27 (46.5%)	**0.002** ^c^	2.5 (1.4–4.5)
Double-embryo transfer	116 (25.5%)	31 (53.5%)	-	-
Clinical pregnancy, single ET *	340 (76.1%)	13 (48.1%)	**0.004** ^c^	3.2 (1.5–7.1)
Clinical pregnancy, double ET *	101 (87.0%)	25 (80.6%)	0.065 ^c^	NS

^c^ Pearson’s Chi-Square Test (Monte Carlo). ET = embryo transfer, CI = confidence interval. * All pregnancy rates are per transfer.

**Table 3:** Pregnancy outcomes between two groups according to the ROC prediction model.

Variable	Biopsy Day 5 (*n* = 456)	Biopsy Day 6 (*n* = 58)	*p*-Value ^c^	Odds Ratio (95% CI)
Age *			**0.016**	2.1 (1.2–3.8)
<35	285 (62.7%)	26 (44.2%)		
≥35	171 (37.3%)	32 (55.8%)		
Clinical pregnancy rates (<35 years) **	226 (79.8%)	10 (56.5%)	**0.016**	3.0 (1.3–7.3)
Clinical pregnancy rates (≥35 years) **	133 (78.0%)	19 (62.1%)	0.098 (NS)	—

^c^ Pearson’s Chi-Square Test (Monte Carlo). CI = confidence interval. * The cut-off point was determined using ROC curve analysis. NS = non-specific. ** All pregnancy rates are per transfer.

## Data Availability

The data that support the findings of this study are available from the corresponding author upon a reasonable request.
